# Bcl6 expression is associated with a distinct immune landscape and spatial transcriptome in COVID-19

**DOI:** 10.1172/jci.insight.189134

**Published:** 2025-09-09

**Authors:** Cloé Brenna, Bernat Bramon Mora, Kalliopi Ioannidou, Julien Bodelet, Mia L. Siebmanns, Simon Burgermeister, Spiros Georgakis, Michail Orfanakis, Yannick D. Muller, Nazanin Sédille, Matthew J. Feinstein, Jon W. Lomasney, Oliver Y. Chén, Giuseppe Pantaleo, Sabina Berezowska, Laurence de Leval, Raphael Gottardo, Constantinos Petrovas

**Affiliations:** 1 Institute of Pathology, Department of Laboratory Medicine and Pathology, and; 2 Biomedical Data Science Center, Lausanne University Hospital (CHUV), Lausanne, Switzerland.; 3 Bioinformatics Platform, Department of Laboratory Medicine and Pathology, CHUV, Lausanne, Switzerland.; 4 Faculty of Biology and Medicine, University of Lausanne, Lausanne, Switzerland.; 5 Service of Immunology and Allergy, Department of Medicine, and; 6 Service of Clinical Chemistry, Department of Laboratory Medicine and Pathology, CHUV, Lausanne, Switzerland.; 7 Feinberg School of Medicine of Northwestern University, Chicago, Illinois, USA.

**Keywords:** Immunology, Infectious disease, COVID-19, T cells, Th1 response

## Abstract

The regulation of follicular (F) and germinal center (GC) immune reactivity in human lymph nodes (LNs), particularly during the acute stages of viral infection, remains poorly understood. We have analyzed lung-draining lymph nodes (LD-LNs) from COVID-19 autopsies using multiplex imaging and spatial transcriptomics to examine the immune landscape with respect to follicular immune reactivity. We identified 3 groups of donors based on the Bcl6 prevalence of their reactive follicles (RFs): RF-Bcl6^no/lo^, RF-Bcl6^int^, and RF-Bcl6^hi^. A distinct B/Tfh immune landscape, associated with increased prevalence of proliferating B cell and Tfh cell subsets, was found in RF-Bcl6^hi^ LD-LNs. The comparison between LD-LNs and subdiaphragmatic (SD) LNs from the same donor revealed a divergent Bcl6 expression between the 2 anatomical sites. LD-LN Bcl6 expression was also associated with a distinct spatial transcriptomic profile. TH1-associated genes/pathways (e.g., *CXCR3*, *STAT5*, TNF signaling) were significantly upregulated in RF-Bcl6^no/lo^ tissues, while the RF-Bcl6^hi^ tissues exhibited significant upregulation of GC-promoting genes/pathways (e.g., *CXCL13*, B-cell receptor signaling). Our findings reveal a heterogeneous F/GC landscape in COVID-19 LD-LNs, highlighting specific molecular targets and pathways that could regulate human F/GC immune dynamics during acute viral infections.

## Abstract 2

We reveal how COVID-19 reshapes immune follicles in lung lymph nodes, identifying distinct molecular pathways that control antibody responses during acute viral infection.

## Graphical abstract

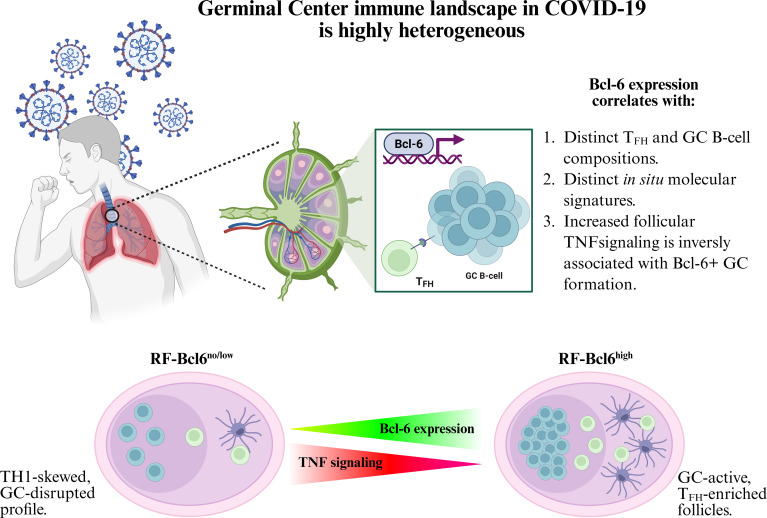

## Introduction

The COVID-19 pandemic, caused by SARS-CoV-2, has significantly affected global health, highlighting the urgent need to understand human immune responses against viral infection (1). Particularly vulnerable groups, such as the elderly and individuals with comorbidities, are at higher risk for severe effects and mortality from COVID-19 and other viral diseases (2). Age-related immune decline, or “immunosenescence,” further compromises the ability to effectively combat infections and respond to vaccination (3).

Lymph nodes (LNs) are crucial for shaping immune responses against pathogens (4). With respect to respiratory infections, thoracic LNs — e.g., Hilar Lymph Nodes (HLNs), located in the lung hilum — are of particular interest as they host antigen-presenting cells (APCs) that capture viral antigens from the lungs, facilitating the activation of naive T cells into effectors to fight the virus. Within the germinal centers (GCs), the coordinated function of Tfh and GC B cells can lead to the development of pathogen-specific B cell responses (5). Tfh cells instruct B cells in undergoing somatic hypermutation (SHM) and affinity maturation, essential for producing high-affinity antibodies and long-term immune memory. Acute COVID-19 infection has been associated with a reduction in GCs and compromised expression of Bcl6, alongside an overexpression of TNFA that could impair the Tfh cell differentiation and development of GC immune reactivity, leading to the generation of long-term B cell memory (6). This impairment may lead to a compromised response upon reinfection or exposure to new variants (7). Understanding the immune landscape of LD-LNs could also provide insights into regional immune responses like effector CD8 T cells in the lungs, the primary site of SARS-CoV-2 infection.

The cellular and molecular mechanisms underlying the development of human F/GC immune reactivity, particularly in acute viral infections, are not well understood. Lack of access to relevant tissues represents a major challenge for such research. Although different viruses may not uniformly induce the same changes in draining LN like COVID-19 (8), using relevant COVID-19 autopsies could provide useful information regarding basic immunological mechanisms mediating the development of F/GC reactivity.

The primary aim of our study was to investigate the F/GC immune reactivity in the context of COVID-19, focusing on possible molecular pathways that could drive the altered F/GC responses in LD-LNs. To this end, we utilized serological measurements, multiplex immunofluorescence (mIF) imaging, and spatial in-situ transcriptomic profiling. Our study investigated Bcl6 expression and its relationship to immune cell composition and transcriptional signatures within the follicular/GC (F/GC). Our findings reveal that differences in Bcl6 expression define distinct reactive follicles (RF) profiles. Mechanistically, our data suggest that increased follicular TH1 signaling in COVID-19 may impair the formation of mature RFs in LD-LNs, potentially contributing to suboptimal antibody responses in infected individuals.

## Results

### COVID-19 infection induces heterogeneous RF profiling in LD-LNs.

**Figure 1:**
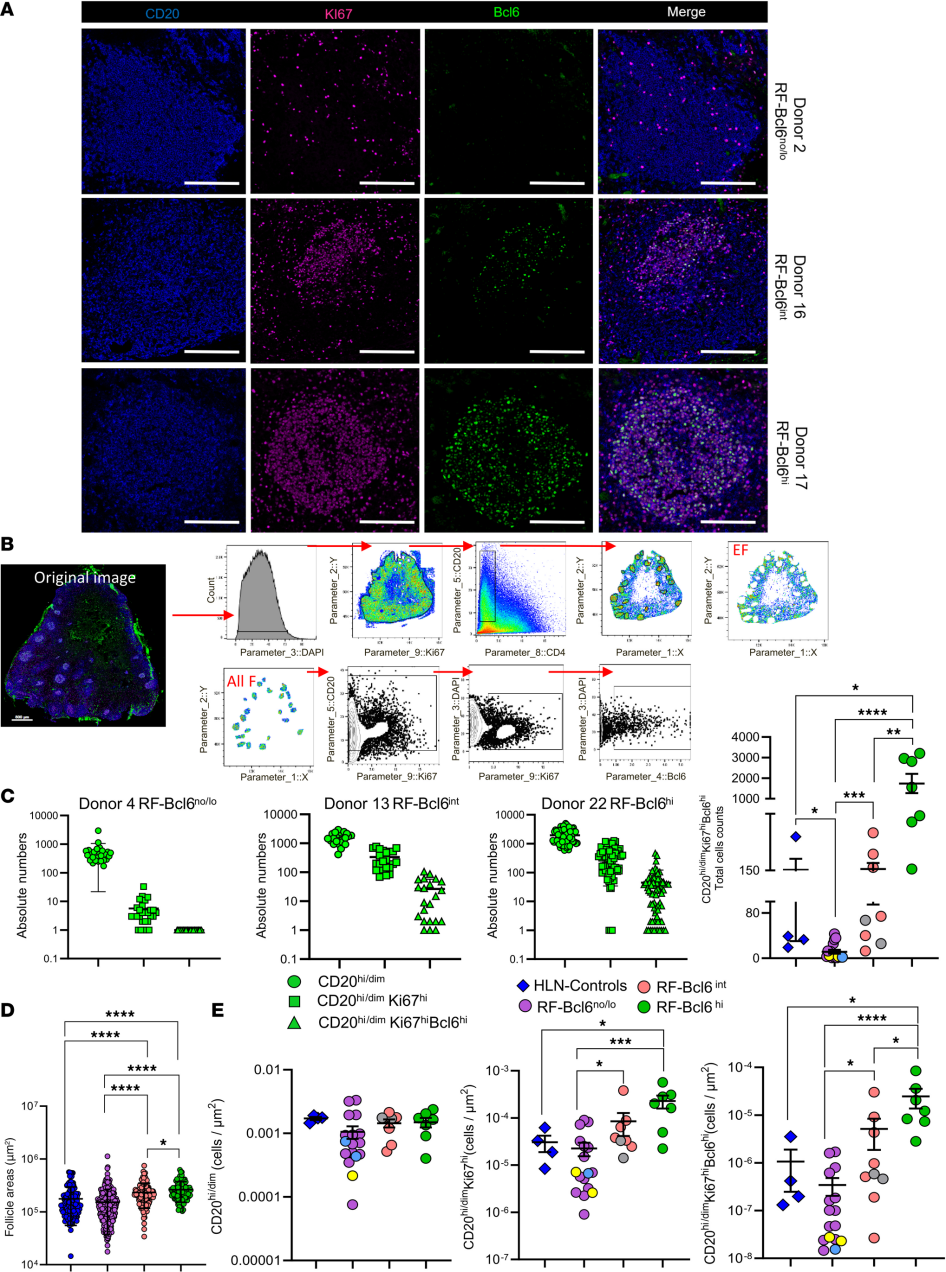
COVID-19 infection induces diverse and heterogeneous reactive follicles in LD-LNs. (**A**) Representative multiplex fluorescence images showing follicular areas from RF-Bcl6^no/lo^ (Donor 2), RF-Bcl6^int^ (Donor 16), and RF-Bcl6^hi^ (Donor 17) tissues with CD20^hi^ (blue), Ki67^hi^ (magenta), Bcl6^hi^ (green), and merged images, highlighting differences in follicular reactivity. Scale bars: 100 μm. (**B**) Histocytometry gating scheme for B cell subset identification based on CD20^hi/dim^ density, with prevalence calculated for all follicles combined (All F). (**C**) Dot plots displaying absolute counts of B cell subsets (CD20^hi/dim^, CD20^hi/dim^Ki67^hi^, CD20^hi/dim^Ki67^hi^Bcl6^hi^) in individual follicles from representative donors — RF-Bcl6^no/lo^ as Donor 4, RF-Bcl6^int^ as Donor 13, RF-Bcl6^hi^ as Donor 22 — illustrating variations in B cell proliferation. The fourth panel summarizes total follicular area CD20^hi/dim^Ki67^hi^Bcl6^hi^ cell counts across the 4 groups (RF-Bcl6^no/lo^, purple; RF-Bcl6^int^, pink; RF-Bcl6^hi^, green), with blue diamonds representing HLN-Controls from non-COVID-19–infected donors. (**D**) Dot plot displaying normalized follicular areas across the 3 RF groups: RF-Bcl6^no/lo^, purple; RF-Bcl6^int^, pink; and RF-Bcl6^hi^, green. Control HLNs from non-COVID-19–infected donors are represented by blue diamonds, highlighting differences in follicular size and maturity. (**E**) Dot plots showing the B cell subset densities (cells/μm²) — CD20^hi/dim^, CD20^hi/dim^ Ki67^hi^, and CD20^hi/dim^Ki67^hi^Bcl6^hi^ — measured in merged follicular areas from each donor. Groups are color-coded as follows: RF-Bcl6^no/lo^, purple; RF-Bcl6^int^, pink; RF-Bcl6^hi^, green; and HLNs from non-COVID-19–infected controls, blue. Light blue dot within the RF-Bcl6^no/lo^ group corresponds to an axillary LN (Donor 25); yellow dot corresponds to para-aortic and mediastinal LNs (Donor 29); and gray dots within the RF-Bcl6^int^ group represent mediastinal LN (Donors 24-a and 24-b). Statistical comparisons were performed using the Mann-Whitney U test; *P ≤ 0.05, **P ≤ 0.01, ***P ≤ 0.001, ****P ≤ 0.0001. Raw *P* values are shown on the graph, and both raw and FDR-adjusted *P* values are reported in Supplemental Table 11.

We started our investigation by analyzing F/GC B cell subsets in LD-LNs from COVID-19–infected individuals from 2 centers (CHUV and Northwestern University) (Table 1 and Supplemental Table 1) by applying a mIF (Supplemental Tables 2 and 3) that allows for the simultaneous detection of CD20, Bcl6, and Ki67 (Figure 1 and Supplemental Figure 1). HLN-Controls from non-COVID-19–infected donors (*n* = 4) were also included in the analysis (Table 1 and Supplemental Figure 2).

Follicular areas, identified by the density of CD20^hi/dim^ cells, were further analyzed for B cell subsets using histocytometry analysis for all combined follicles (total follicular area [All F]) (Figure 1B). Visual inspection of raw mIF images, along with quantitative analysis of Bcl6^hi^ B cell counts, either per follicular area or total follicular area per tissue, revealed a high heterogeneity of follicular Bcl6 expression within individual LNs, with mixed levels observed among follicles (Figure 1C; Supplemental Figure 3, A–C; and Supplemental Figure 4A).

Based on the Bcl6 profiling, 3 distinct tissue groups were identified: RF-Bcl6^no/lo^ (mean ± SD: 48.3 ± 23.9 follicles analyzed), RF-Bcl6^int^ (50.8 ± 24.9 follicles analyzed), and RF-Bcl6^hi^ (66.3 ± 25.2 follicles analyzed) (Figure 1C; Supplemental Figure 3, A–C; and Supplemental Figure 4A). No significant difference in the number of follicles analyzed among the 3 groups was observed, as assessed by a Mann-Whitney *U* test (Supplemental Table 4).

The time interval between death and autopsy procedure did not differ significantly between groups (CHUV samples), with an average of 19.86 ± 12.26 hours (RF-Bcl6^no/lo^), 35.53 ± 24.70 hours (RF-Bcl6^int^), and 33.22 ± 26.72 hours (RF-Bcl6^hi^) (Supplemental Table 5). A Mann-Whitney *U* test showed no significant differences between death and autopsy procedure between RF-Bcl6^no/lo^ and RF-Bcl6^int^ (*P* = 0.1451), RF-Bcl6^no/lo^ and RF-Bcl6^hi^ (*P* = 0.3283), or RF-Bcl6^int^ and RF-Bcl6^hi^ (*P* > 0.9999), confirming no influence on RF-Bcl6 expression patterns. Furthermore, no consistent association was observed between immunosuppressive treatments and Bcl6 expression levels across donors, suggesting that local immune dynamics, rather than systemic immunosuppression alone, may drive the heterogeneity in RF-Bcl6 groups (Table 2). Notably, the RF-Bcl6^no/lo^ group displayed Ki67 expression, indicating active proliferation and confirming these as RF rather than primary or resting follicles.

Analysis of RF morphological features revealed that individual RFs exhibited a significantly larger area in both RF-Bcl6^int^ and RF-Bcl6^hi^ groups when compared with RF-Bcl6^no/lo^ and HLN-Controls from non-COVID-19–infected donors (Figure 1D). Although an increase in follicular size can be observed in various reactive contexts, our data indicate that this enlargement is particularly associated with higher Bcl6 expression in the setting of COVID-19, potentially reflecting follicular expansion and/or enhanced maturation of GC responses in the former 2 groups. No significant differences were observed in other morphological features analyzed.

Next, the cell densities (normalized cell counts per μm²) of specific B cell subsets were calculated for all available tissues. Despite similar cell densities of bulk CD20^hi/dim^ B cells across the HLN-Controls and the 3 groups, RF-Bcl6^int^ and especially RF-Bcl6^hi^ tissues were characterized by significantly higher densities of proliferating Ki67^hi^ and CD20^hi/dim^Ki67^hi^Bcl6^hi^ B cells (Figure 1E). A similar profile was observed when the frequency of B cell subsets was calculated (Supplemental Figure 4A).

Then, the pattern of the follicular DC (FDC) network, as an additional surrogate of mature RFs, was evaluated (Supplemental Figure 4B). We found a diverse expression of FDC in most RFs from both RF-Bcl6^no/lo^ and RF-Bcl6^hi^ tissues. However, the FDC staining pattern varied between the 2 groups, with a connected network pattern more frequently observed in RF-Bcl6^hi^ tissues, while unconnected FDC^+^ events, a sign of less active and mature RFs, were commonly seen in RF-Bcl6^no/lo^ tissues (Supplemental Figure 4B). Altogether, our data suggest that RFs, as indicated by the cell densities of Ki67^hi^ and Bcl6^hi^ B cells, in LD-LNs from COVID-19–infected individuals are highly diverse and heterogeneous.

We next assessed the relationship between RF-Bcl6 expression and serological markers taken during hospitalization (CHUV donors) using a mixed linear regression model to account for repeated measures and donor variability (Supplemental Table 6). A trend toward higher CRP levels was observed in RF-Bcl6^no/lo^ compared with RF-Bcl6^hi^ (β = –61.56, *P* = 0.257), though leukocyte and lymphocyte counts did not differ significantly (Supplemental Table 6). We then examined whether RF-Bcl6 expression varied in relation to the estimated time after infection, as estimated by the duration of hospitalization. Given the cross-sectional nature of the study, this analysis does not infer longitudinal changes within individuals but assesses whether longer hospitalization is associated with differences in Bcl6 expression. No significant changes in RF-Bcl6 expression were observed over time in RF-Bcl6^no/lo^ (*P* = 0.927) or RF-Bcl6^hi^ (*P* = 0.903) groups (Supplemental Figure 4C and Supplemental Table 7), suggesting that Bcl6 expression does not progressively increase during hospitalization. However, a significant decline was observed in the RF-Bcl6^int^ group (β = –60.175, *P* = 0.013) (Supplemental Table 7), while at the general level only a modest, nonsignificant decrease was detected (β = –0.00446, *P* = 0.179) (Supplemental Table 7). Overall, our data suggest that the heterogeneity of LD-LNs RF-Bcl6 expression likely reflects the intrinsic capacity of the immune system to mount mature GC-RFs rather than a direct consequence of hospitalization duration or post-infection timing.

### RF-Bcl6^hi^ LD-LNs harbor higher cell densities of proliferating CD57^hi^ Tfh cell.

**Figure 2:**
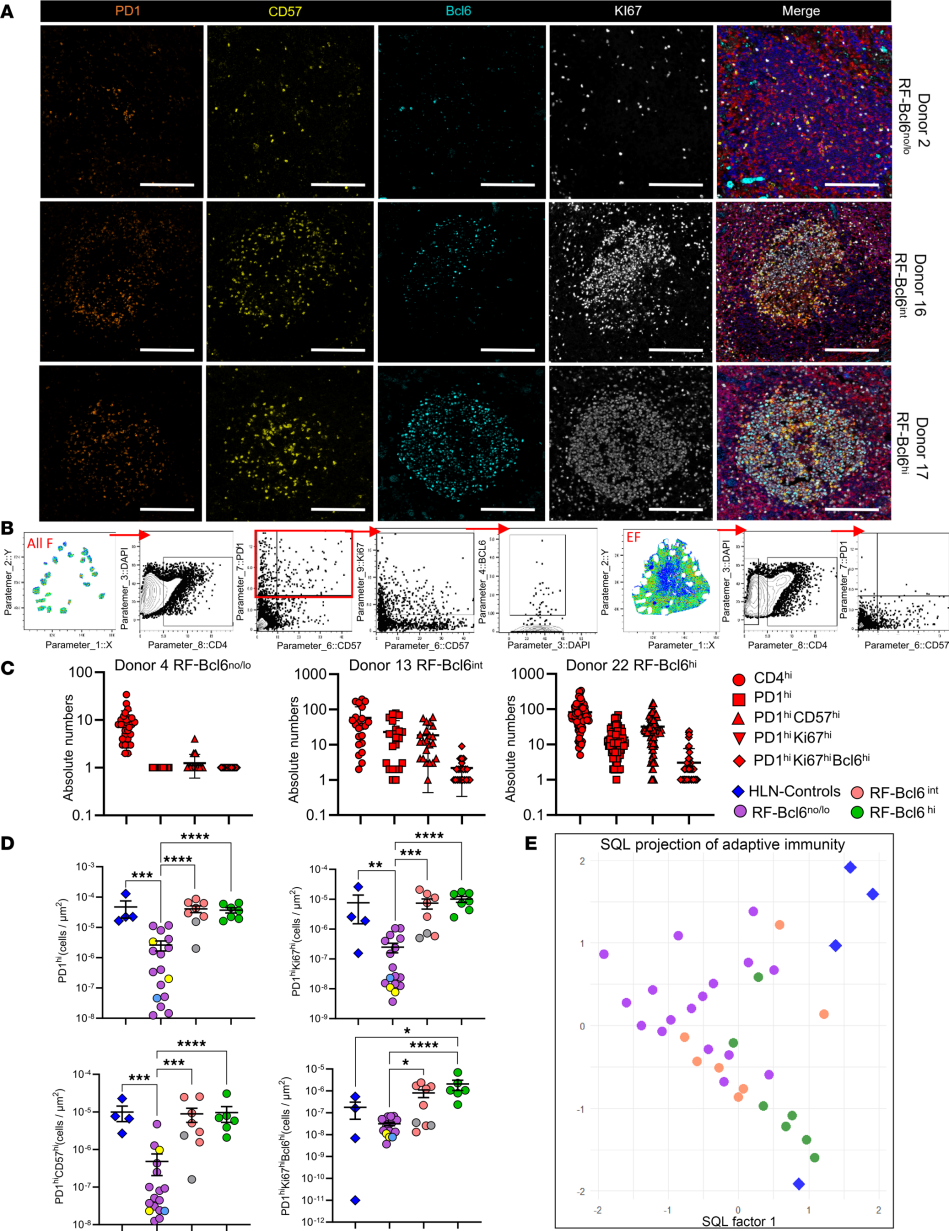
RF-Bcl6^hi^ LD-LNs harbor higher densities of proliferating Bcl6^hi^ and CD57^hi^ Tfh cells. (**A**) Representative fluorescence images of follicular areas from RF-Bcl6^no/lo^ (Donor 2), RF-Bcl6^int^ (Donor 16), and RF-Bcl6^hi^ (Donor 17) tissues showing PD1^hi^ (orange), CD57^hi^ (yellow), Bcl6^hi^ (cyan), Ki67^hi^ (white), and merged images, highlighting Tfh cell subset differences. Scale bars: 100 μm. (**B**) Histocytometry gating scheme for CD4 T cell subset identification within CD20^hi/dim^ follicular areas, with prevalence calculated for all follicles combined (All F). (**C**) Dot plots showing absolute counts of Tfh cell subsets (PD1^hi^, PD1^hi^CD57^hi^, PD1^hi^Ki67^hi^, PD1^hi^Ki67^hi^Bcl6^hi^) per follicle from representative donors (RF-Bcl6^no/lo^, Donor 4; RF-Bcl6^int^, Donor 13; RF-Bcl6^hi^, Donor 22). (**D**) Dot plots of normalized Tfh cell subset densities (cells/μm²) — PD1^hi^, PD1^hi^ Ki67^hi^, PD1^hi^CD57^hi^Bcl6^hi^, and PD1^hi^Ki67^hi^Bcl6^hi^ — measured in merged follicular areas from each donor. Groups are color-coded as follows: RF-Bcl6^no/lo^ (purple), RF-Bcl6^int^ (pink), RF-Bcl6^hi^ (green), and HLNs from non-COVID-19–infected controls (blue). Light blue dot within the RF-Bcl6^no/lo^ group corresponds to an axillary LN (Donor 25); yellow dot corresponds to para-aortic and mediastinal LNs (Donor 29); gray dots within the RF-Bcl6^int^ group represent mediastinal LN (Donors 24-a and 24-b). (**E**) SQL clustering projection of B cell and Tfh cell subsets based on the expression of CD20^hi/dim^, CD20^hi/dim^Ki67^hi^, CD20^hi/dim^Ki67^hi^Bcl6^hi^, CD4^hi^, PD1^hi^, PD1^hi^Ki67^hi^, PD1^hi^CD57^hi^, and PD1^hi^Ki67^hi^Bcl6^hi^. Data points are color-coded by RF group: RF-Bcl6^no/lo^ (purple), RF-Bcl6^int^ (pink), and RF-Bcl6^hi^ (green). HLNs from non-COVID-19–infected control donors are shown as blue diamonds. The projection displays SQL Factor 1 versus SQL Factor 3, which capture the main variance across immune cell phenotypes and best separate the groups. Mann-Whitney *U* test; *P ≤ 0.05, **P ≤ 0.01, ***P ≤ 0.001, ****P ≤ 0.0001. Raw *P* values are shown on the graph, and both raw and FDR-adjusted *P* values are reported in Supplemental Table 11.

Given the essential role of Tfh cells in the formation and maintenance of GC-RFs (5), we aimed to analyze their in situ cell densities across the 3 aforementioned groups. Different Tfh cell subsets were identified based on the expression of PD1, Bcl6, Ki67, and CD57 expression, a marker that defines a distinct Tfh cells subset (9, 10) (Figure 2 and Supplemental Figure 4D). We noticed an inconsistency in the coexpression of CD57 and CD4 in RFs among individuals, possibly reflecting a downregulation of the CD4 receptor, similar to the pattern observed for CD3 expression (9) in highly differentiated Tfh cells. Given this characteristic, we refined our gating strategy to avoid bias by identifying Tfh cells as PD1^hi^ and PD1^hi^CD57^hi^ (Figure 2B). To set relevant cut-off values, we used PD1 and CD57 expression levels in extrafollicular (EF) CD4 T cells as a reference and applied these thresholds across the entire F region (Figure 2B).

The calculation of absolute numbers of Bcl6^+^ Tfh cells across individual RFs revealed a distribution pattern similar to that observed for B cells (Figure 2C; Supplemental Figure 3, A–C; and Supplemental Figure 4A). A significantly higher cell density of PD1^hi^, PD1^hi^Ki67^hi^, PD1^hi^CD57^hi^, and PD1^hi^Ki67^hi^Bcl6^hi^ cells in RF-Bcl6^int^ and RF-Bcl6^hi^ compared with RF-Bcl6^no/lo^ tissues was observed (Figure 2D), suggesting an association between Tfh cells differentiation and Bcl6-expression in these RFs. Unlike B cells, a significant difference between HLN-Controls from non-COVID-19–infected and RF-Bcl6^hi^ LNs was found only for the PD1^hi^Ki67^hi^Bcl6^hi^ cells (Figure 2D).

A similar trend was observed when analyzing the frequency of Tfh cell subsets (Supplemental Figure 4E). To further explore the relationship between Bcl6 expression and follicular immune cell composition, we applied the statistical quantile learning (SQL) method (11) for clustering analysis based on B cells and Tfh cells measurements. The SQL projection of adaptive immunity (Figure 2E) revealed a gradual transition in immune cell clustering that aligns with increasing Bcl6 expression.

Altogether, our data indicate that the expression of Bcl6 in LD-LNs from COVID-19 autopsies reflects a spectrum of follicular immune reactivity.

### The increasing prevalence of Bcl6 is associated with an altered spatial distribution of RF immune cell subsets.

Next, we analyzed the spatial distribution of key immune cell subsets within RFs in the RF-Bcl6^int^ and RF-Bcl6^hi^ groups by applying linear regression models and relevant algorithms.

**Figure 3:**
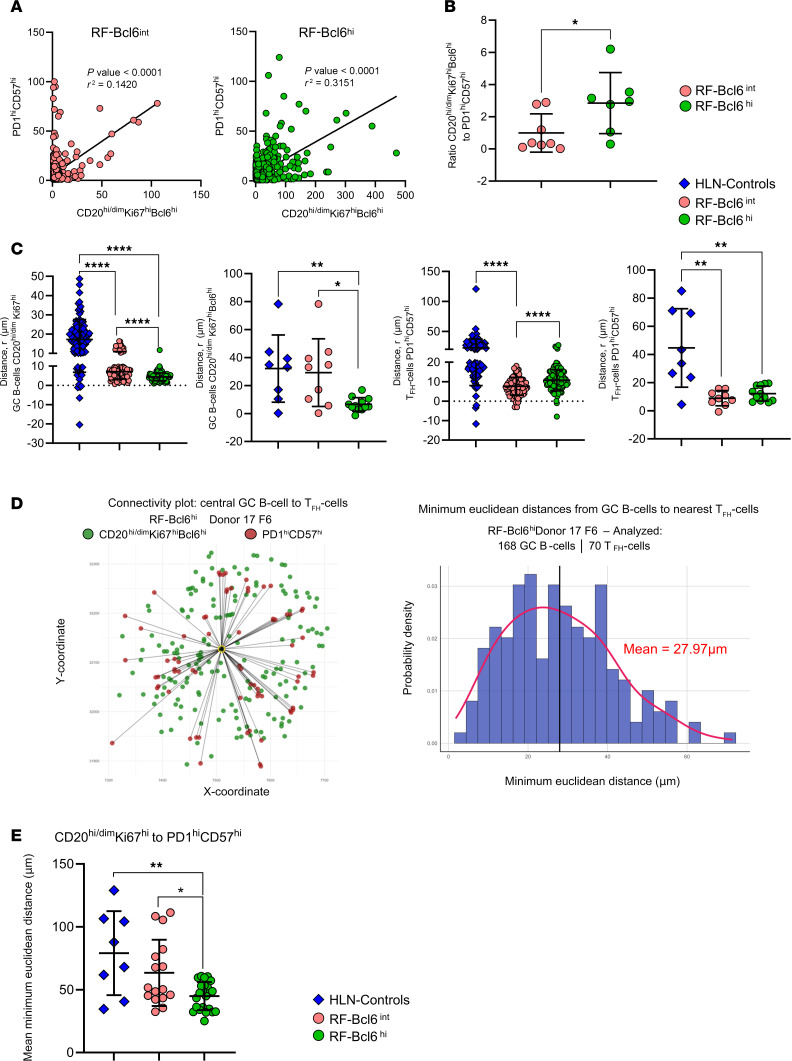
Increasing Bcl6 expression is associated with altered spatial distribution of RF immune cell subsets. (**A**) Correlation between PD1^hi^CD57^hi^ Tfh cells and CD20^hi/dim^Ki67^hi^Bcl6^hi^ B cells across follicles in LD-LNs. Left panel: RF-Bcl6int donors (pink). Right panel: RF-Bcl6^hi^ donors (green). *R*² and *P* values are reported. (**B**) Ratio of CD20^hi/dim^Ki67^hi^Bcl6^hi^ B cells to PD1^hi^CD57^hi^ Tfh cells in RF-Bcl6^int^ (pink) versus RF-Bcl6^hi^ (green) donors. (**C**) Net area under the G-function curve (AUCabove – AUCbelow) by distance (r, μm), comparing spatial organization of GC B cells and Tfh cells subsets across groups (tissues from CHUV were analyzed). (**D**) Left panel: Connectivity plot from Donor 17-Follicle 6 (RF-Bcl6^hi^) showing spatial links from the most central GC B cell (yellow) to Tfh cells (green). Right panel: Histogram of minimum Euclidean distances from Donor 17-Follicle 6 (RF-Bcl6^hi^). (**E**) Dot plot of mean minimum Euclidean distances between CD20^hi/dim^Ki67^hi^ B cells and PD1^hi^CD57^hi^ Tfh cells in HLN-Controls (blue), RF-Bcl6int (pink), and RF-Bcl6^hi^ (green). Each point represents one follicle. Mann-Whitney *U*; *P ≤ 0.05, **P ≤ 0.01, ****P ≤ 0.0001. Raw *P*-values are shown on the graph, and both raw and FDR-adjusted *P*-values are reported in Supplemental Table 11.

A significant positive correlation was found between PD1^hi^CD57^hi^ Tfh cells and CD20^hi/dim^Ki67^hi^Bcl6^hi^ B cells in both the RF-Bcl6^hi^ (*R*^2^ = 0.32, *P* < 0.001) and RF-Bcl6^int^ (*R*^2^ = 0.14, *P* < 0.001) groups when individual follicles were analyzed (Figure 3A). Notably, this correlation was stronger and more consistently significant for individual donors in the RF-Bcl6^hi^ group compared with RF-Bcl6^int^ (Supplemental Figure 5, A and B).

Furthermore, the ratio of CD20^hi/dim^Ki67^hi^Bcl6^hi^ B cells to PD1^hi^CD57^hi^ Tfh cells was significantly higher in RF-Bcl6^hi^ compared with RF-Bcl6^int^ (Figure 3B), suggesting an increased likelihood of productive B/T cell interactions in RF-Bcl6^hi^ follicles.

To further investigate the spatial dynamics of GC B cell and Tfh cell interactions, we extracted the spatial coordinates of CD20^hi/dim^Ki67^hi^, CD20^hi/dim^Ki67^hi^Bcl6^hi^, PD1^hi^, and PD1^hi^CD57^hi^ cells in RFs containing at least 20 cells per phenotype. We then applied the G-function, a spatial statistical method that quantifies cell clustering or dispersion by comparing observed spatial distributions to a random Poisson model using net AUC as a metric (Figure 3C and Supplemental Figure 6A). We found that CD20^hi/dim^Ki67^hi^ and CD20^hi/dim^Ki67^hi^Bcl6^hi^ B cells exhibited a significantly less scattered (more clustered) distribution in RF-Bcl6^int^ tissue compared with RF-Bcl6^hi^ (Figure 3C). In contrast, PD1^hi^ Tfh cells displayed the opposite pattern, with a more dispersed distribution in RF-Bcl6^int^ tissue (Figure 3C). Furthermore, RF-Bcl6^hi^ follicles exhibited a significantly lower clustering profile of both B cells and Tfh cell subsets compared with HLN-Controls of non-COVID-19–infected patients (Figure 3C).

Our distance matrix analysis (an example is shown in Figure 3D) revealed a comparable mean minimum Euclidean distance between proliferating CD20^hi/dim^Ki67^hi^ B cells and PD1^hi^CD57^lo^ Tfh cells (Supplemental Figure 6B) and between GC B cells CD20^hi/dim^Ki67^hi^Bcl6^hi^, and PD1^hi^CD57^hi^ Tfh cells (Supplemental Figure 6B). However, a significantly shorter distance between CD20^hi/dim^Ki67^hi^ B cells and PD1^hi^CD57^hi^ Tfh cells was observed in RF-Bcl6^hi^ compared with RF-Bcl6^int^ (*P* = 0.0432) and HLN-Controls (*P* = 0.0046) (Figure 3E), suggesting a higher possibility for direct B/Tfh interactions in RF-Bcl6^hi^ LD-LNs.

Altogether, our data suggest that increasing Bcl6 expression in RFs is associated with enhanced spatial proximity and interaction between proliferating B cells and highly differentiated Tfh cells.

### COVID-19 infection reveals a disconnection in RF immunoreactivity between LD-LNs and matched distal subdiaphragmatic LNs.

**Figure 4:**
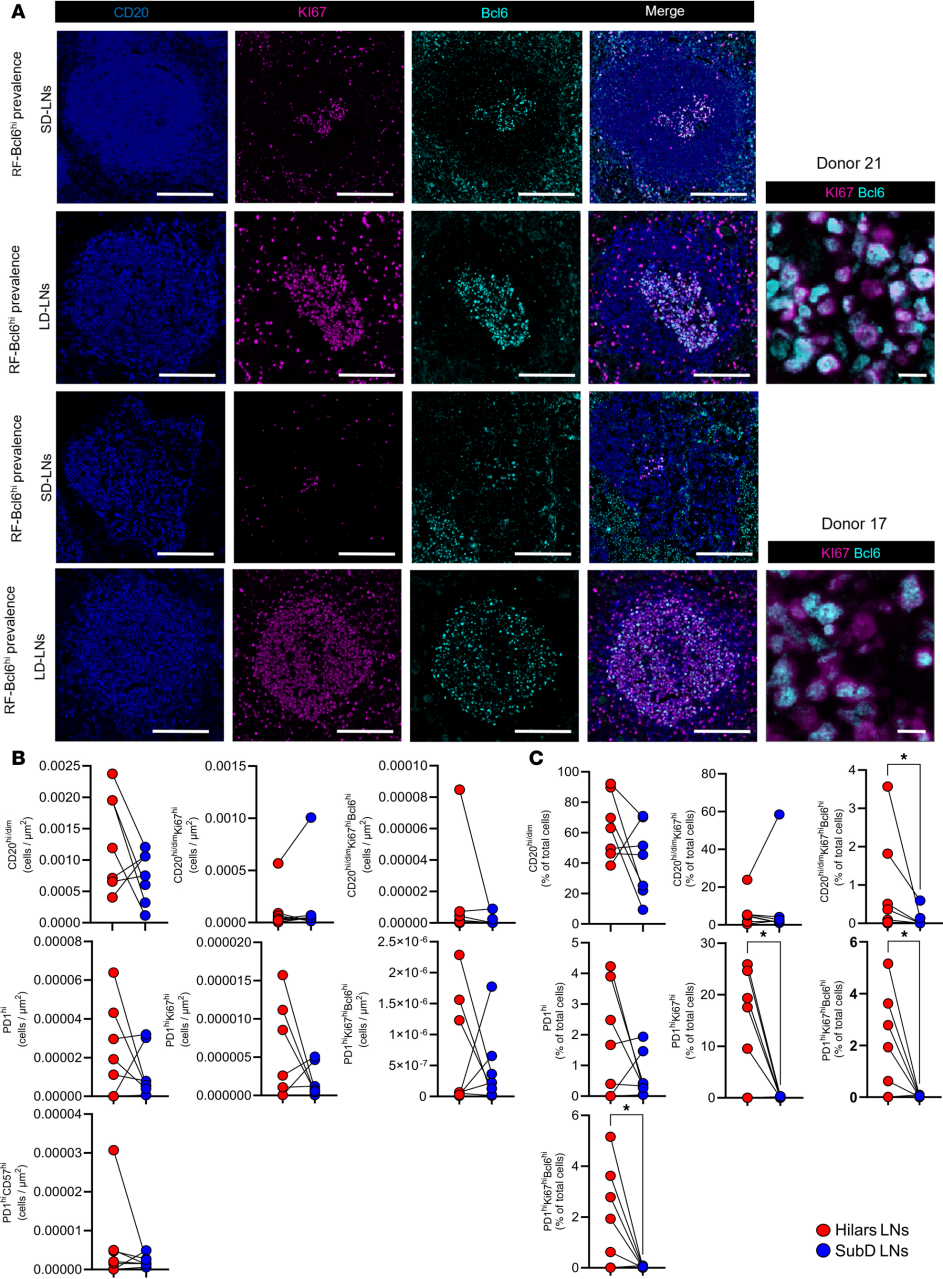
COVID-19 infection reveals a disconnection in F/GC immunoreactivity between LD-LNs and matched distal subdiaphragmatic LNs. (**A**) Representative fluorescence images of follicular areas from subdiaphragmatic and matched LD-LNs in 2 RF-Bcl6^hi^ donors. Upper panel: Donor 21. Lower panel: Donor 17. Staining shows CD20^hi^ (blue), Ki67^hi^ (magenta), Bcl6^hi^ (cyan), along with merged composite images. Scale bars: Original magnification, 100 μm (original); 10 μm (zoomed area). (**B**) Dot plots showing normalized densities of B cells and CD4 T cells (cells/μm²) in subdiaphragmatic (blue) and matched LD-LNs (red). (**C**) Dot plots showing frequencies of B cell and CD4 T cell subsets (% of total cells) in subdiaphragmatic (blue) and matched LD-LNs (red). Tissues from CHUV were analyzed. Wilcoxon test; *P ≤ 0.05.

We analyzed lung-draining LNs and subdiaphragmatic LNs (SD-LNs; serving as “internal controls”) from the same donors (CHUV site) (Figure 4, A–C, Supplemental Table 8, and Supplemental Figure 6C) to investigate whether COVID-19 infection leads to a generalized RF Bcl6 immunoreactivity. The adaptive immunity panel (Supplemental Tables 2 and 3) was applied to SD-LNs (Figure 4A).

Overall, we observed a tendency toward lower cell densities of B cell and Tfh cell subsets in SD-LNs, with a significant decrease in CD20^hi/dim^Ki67^hi^Bcl6^hi^ cells compared with their LD-LN counterparts (Figure 4B). Consistent with this profile, SD-LNs exhibited significantly lower frequencies of CD20^hi/dim^Ki67^hi^Bcl6^hi^, PD1^hi^Ki67^hi^, and PD1^hi^Ki67^hi^Bcl6^hi^ cells compared with LD-LNs (Figure 4C).

Our data suggest that the heightened RF activity seen in LD-LNs possibly represents a localized adaptation in lung-draining lymphoid tissues rather than a generalized systemic response.

### The RF-Bcl6^hi^ tissues are characterized by a distinct in situ follicular transcriptomic profile.

**Figure 5:**
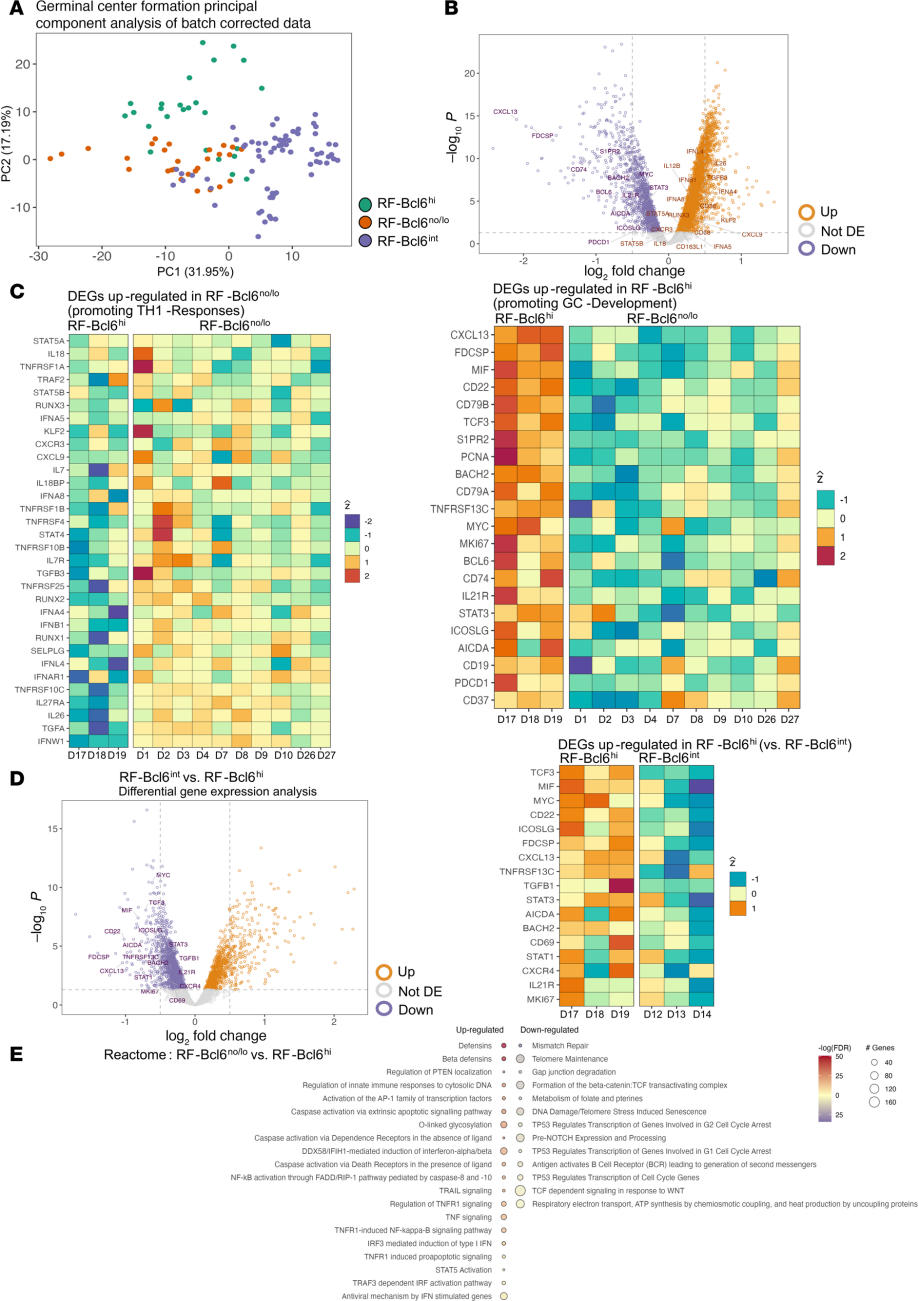
Distinct in situ transcriptomic profiles are associated with RF-Bcl6^hi^ follicles in LD-LNs. (**A**) Principal component analysis (PCA) of gene expression following normalization and batch correction. Samples are color-coded by group — RF-Bcl6^hi^ (green), RF-Bcl6^int^ (orange), and RF-Bcl6^no/lo^ (purple). (**B**) Volcano plot showing differentially expressed genes between RF-Bcl6^no/lo^ and RF-Bcl6^hi^ individuals. Orange dots indicate genes upregulated in RF-Bcl6^no/lo^, purple dots in RF-Bcl6^hi^, and gray dots denote nonsignificant genes. (**C**) Heatmaps of differentially expressed genes (DEGs) between RF-Bcl6^no/lo^ and RF-Bcl6^hi^ individuals. Left panel: DEGs associated with TH1 responses, upregulated in the RF-Bcl6^no/lo^ group. Right panel: DEGs related to germinal center (GC) development, upregulated in the RF-Bcl6^hi^ group. Colors represent Z-scored expression values. (**D**) Left panel: Volcano plot showing differentially expressed genes (DEGs) between RF-Bcl6^int^ and RF-Bcl6^hi^ individuals. Orange dots indicate genes significantly upregulated in RF-Bcl6^int^, while purple dots represent those upregulated in RF-Bcl6^hi^. Right panel: Heatmap displaying DEGs upregulated in RF-Bcl6^hi^ group. Colors indicate standardized Z-scores, reflecting relative gene expression levels across samples. (**E**) Dot plot of Reactome pathways significantly enriched in RF-Bcl6^no/lo^ versus RF-Bcl6^hi^ individuals. Dot size indicates the number of differentially expressed genes that are upregulated (left column) or downregulated (right column) in RF-Bcl6^no/lo^. Dot color represents the –log (FDR), corresponding to the negative logarithm of the FDR.

The GeoMx platform was applied to RF areas covering the 3 groups (CHUV site), using normalized and batch-corrected data for transcriptomic analysis (Supplemental Figure 7, A and B). Principal component analysis (PCA) revealed distinct transcriptional profiles based on Bcl6 expression, suggesting molecular differences in GC reactivity across the groups (Figure 5A).

Several differentially expressed genes (DEGs) emerged from the RF-Bcl6^no/lo^ versus RF-Bcl6^hi^ comparison (Figure 5B), highlighting functional divergence in immune pathways. In RF-Bcl6^hi^ donors, genes linked to GC-development and B cell activation (e.g., *BCL6*, *AICDA*, *IL21R*, *CXCL13*, *STAT3*, *CD79A*, *MYC*, *MKI67*) were upregulated, reinforcing their role in sustaining GC activity (Figure 5C). Conversely, in RF-Bcl6^no/lo^ donors, genes associated with a TH1-driven immune response and inflammation (e.g., *STAT4*, *TNFRSF10B*, *TNFRSF1A*, *CXCR3*, *TGFB3*, *IL7*, *IFNB1*, *RUNX1*) were significantly upregulated, suggesting a shift toward a proinflammatory and interferon-driven environment (Figure 5C).

Analysis of PCA projection of the ROIs based on the selected gene sets revealed a more clustered expression of TH1-related genes in RF-Bcl6^no/lo^ tissues, whereas GC-associated genes exhibited a more scattered profile in RF-Bcl6^hi^ tissues (Supplemental Figure 7C and Supplemental Figure 8A). The data indicate a more homogeneous distribution of TH1-related genes — and presumably of associated signaling pathways — among RFs within a given tissue, in contrast to the more heterogeneous pattern observed for GC-development–related genes.

Comparing RF-Bcl6^int^ versus RF-Bcl6^hi^ tissues (Figure 5D), we found a significant upregulation of genes favoring Tfh/GC formation (e.g., *CXCL13*, *CD22*, *MIF*, *STAT3*, *AICDA*) in RF-Bcl6^hi^ tissues (Figure 5D). Examining expression profiles across all groups, GC-promoting genes (e.g., *AICDA*, *BCL6*, *IL21R*, *CD19*) showed a progressive upregulation from RF-Bcl6^no/lo^ to RF-Bcl6^hi^ (Supplemental Figure 8B). This was further supported by increased expression of *CXCL13*, *S1PR2*, and *PCNA*, key markers of GC organization and proliferation. Meanwhile, TH1-associated genes (e.g., *STAT4*, *TNFRSF10B*, *TNFRSF1B*) were consistently downregulated in RF-Bcl6^hi^ compared with RF-Bcl6^no/lo^, whereas RF-Bcl6^int^ exhibited an intermediate profile, with some TH1-related genes (e.g., *CXCR3*, *IFNB1*) maintaining moderate expression (Supplemental Figure 8B).

Pathway enrichment analysis revealed upregulation of TNF-related (e.g., TNFR1 Signaling, NF-κβ, TRAIL Signaling) and IFN pathways (e.g., α/β Interferon Signaling) in RF-Bcl6^no/lo^ tissues, suggesting a sustained inflammatory response in these follicles (Figure 5E). Conversely, RF-Bcl6^hi^ tissues showed enrichment of pathways related to metabolism, DNA repair, and immune activation (e.g., Respiratory Electron Transport, ATP Synthesis, Mismatch Repair), suggesting enhanced GC maintenance and cellular homeostasis. Furthermore, the RF-Bcl6^hi^ group showed upregulation of RF-development pathways (e.g., IL-4 signaling, IL-6 signaling, detoxification of reactive oxygen species) compared with RF-Bcl6^int^ (Supplemental Figure 8C). Overall, our findings suggest that RF-Bcl6 expression in COVID-19 LD-LNs is linked to distinct in situ molecular profiles.

### B cells and Tfh cells differentially influence TNF signaling depending on RF-Bcl6 expression.

**Figure 6:**
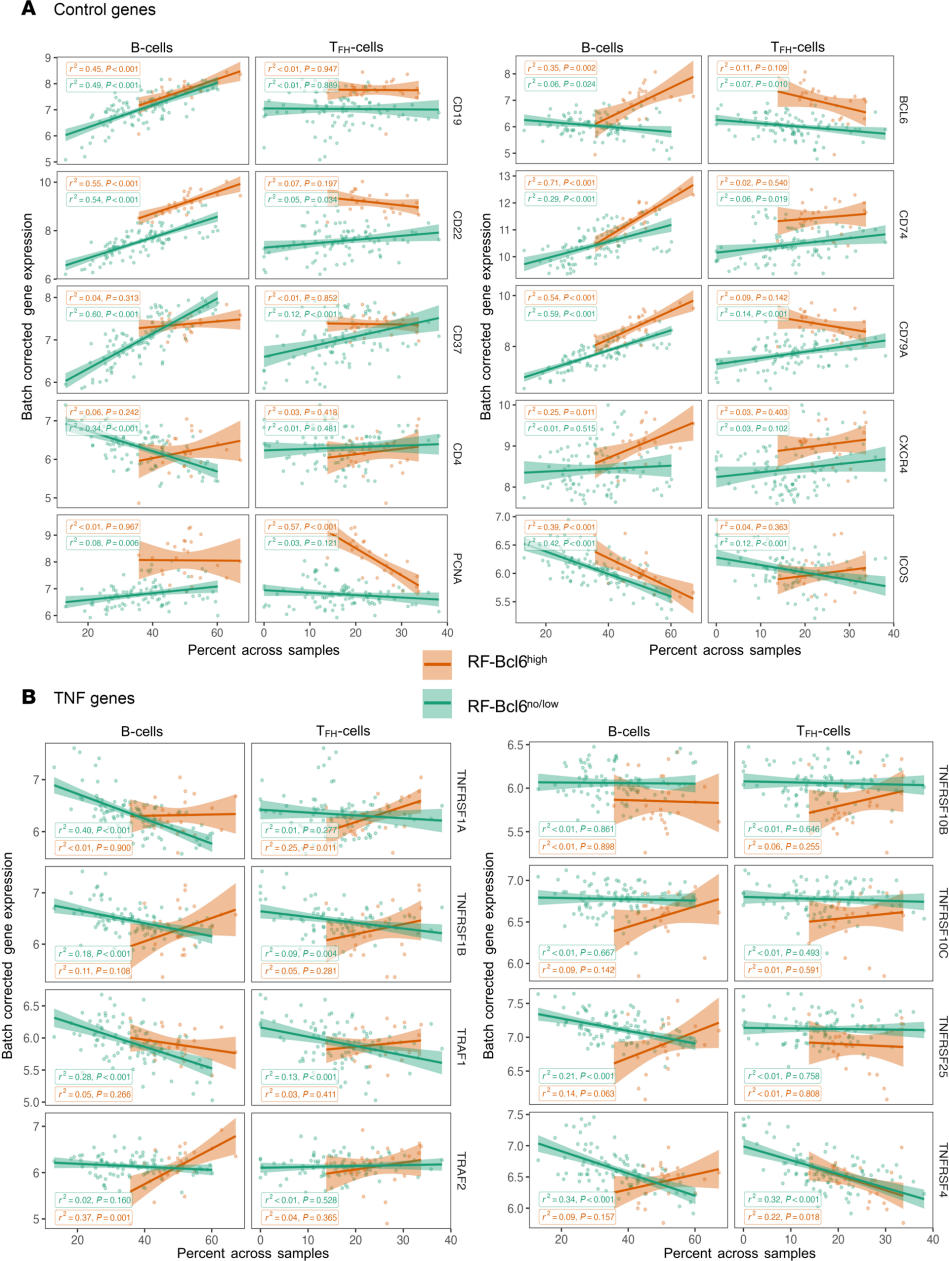
Association between TNF-family gene expression and follicular B cells or Tfh cells. (**A**) Dot plots illustrating the correlation between the expression levels of selected control genes — chosen for their established cell type specificity — and the inferred percentages of B cells or Tfh cells obtained through transcriptomic cell deconvolution analysis. The *y* axis displays batch-corrected and normalized gene expression values, allowing for cross-sample comparability. Each data point represents an individual follicle (region of interest, ROI) derived from tissue samples classified as either RF-Bcl6^no/lo^ (green) or RF-Bcl6^hi^ (orange), enabling visualization of expression trends across reactive follicle subtypes. (**B**) Dot plots showing the correlation between the expression levels of TNF family genes and the inferred proportions of B cells or Tfh cells, as determined by transcriptomic deconvolution. The *y* axis represents batch-corrected and normalized gene expression values, facilitating robust comparison across samples. Each point corresponds to a distinct follicle (region of interest, ROI) originating from either the RF-Bcl6^no/lo^ group (green) or the RF-Bcl6^hi^ group (orange), allowing assessment of how TNF-related signaling may associate with specific immune cell populations. For each correlation, the coefficient of determination (*R*²) and corresponding *P* value are reported, indicating the strength and significance of the observed associations.

To further characterize the cellular composition and gene expression profiles in RF-Bcl6^hi^ and RF-Bcl6^no/lo^ tissues, we conducted a cell deconvolution analysis using single-cell RNA-Seq data from HIV LNs as a reference. This approach enabled us to evaluate the relative abundance of immune cell populations across tissue groups. Our analysis examined the relationship between gene expression and the relative proportions of specific cell types (B cells or Tfh cells). The expression of control genes (with known expression profiles) showed a significant positive association between B cells and B cell–related genes (e.g., *CD19*, *CD22*, *CD74*, *CD79A*) as well as between Tfh cells and (e.g., *ICOS*), supporting the validity of our pipeline (Figure 6A).

We observed heterogeneous associations between B cells, Tfh cells, and TNF-family genes (Figure 6B), which were differentially expressed between RF-Bcl6^no/lo^ and RF-Bcl6^hi^ samples (Figure 6B). In the RF-Bcl6^no/lo^ group, a strong negative association was detected between B cells and several genes (e.g., *TNFRSF1A/TNF-R1*, *TNFRSF1B/TNF-R2*, *TRAF1*, *TNFRSF4/CD134*, and *TNFRSF25/DR3*) (Figure 6B). This negative association was less pronounced for Tfh cells (Figure 6B). In contrast, the RF-Bcl6^hi^ group showed a trend toward a positive association between several genes and B cells, reaching significance for *TRAF2* (Figure 6B), as well as between Tfh cells and TNFRSF1A/TNF-R1 (Figure 6B).

Together, these findings suggest that differences in TNF-signaling gene expression across RF-Bcl6 groups may reflect distinct immune microenvironments, with altered B cell and Tfh cell dynamics potentially contributing to the divergent follicular responses observed in RF-Bcl6^hi^ versus RF-Bcl6^no/lo^ tissues.

### RF-Bcl6^no/lo^ tissues are characterized by a distinct in situ follicular macrophage–associated gene-expression profile.

**Figure 7:**
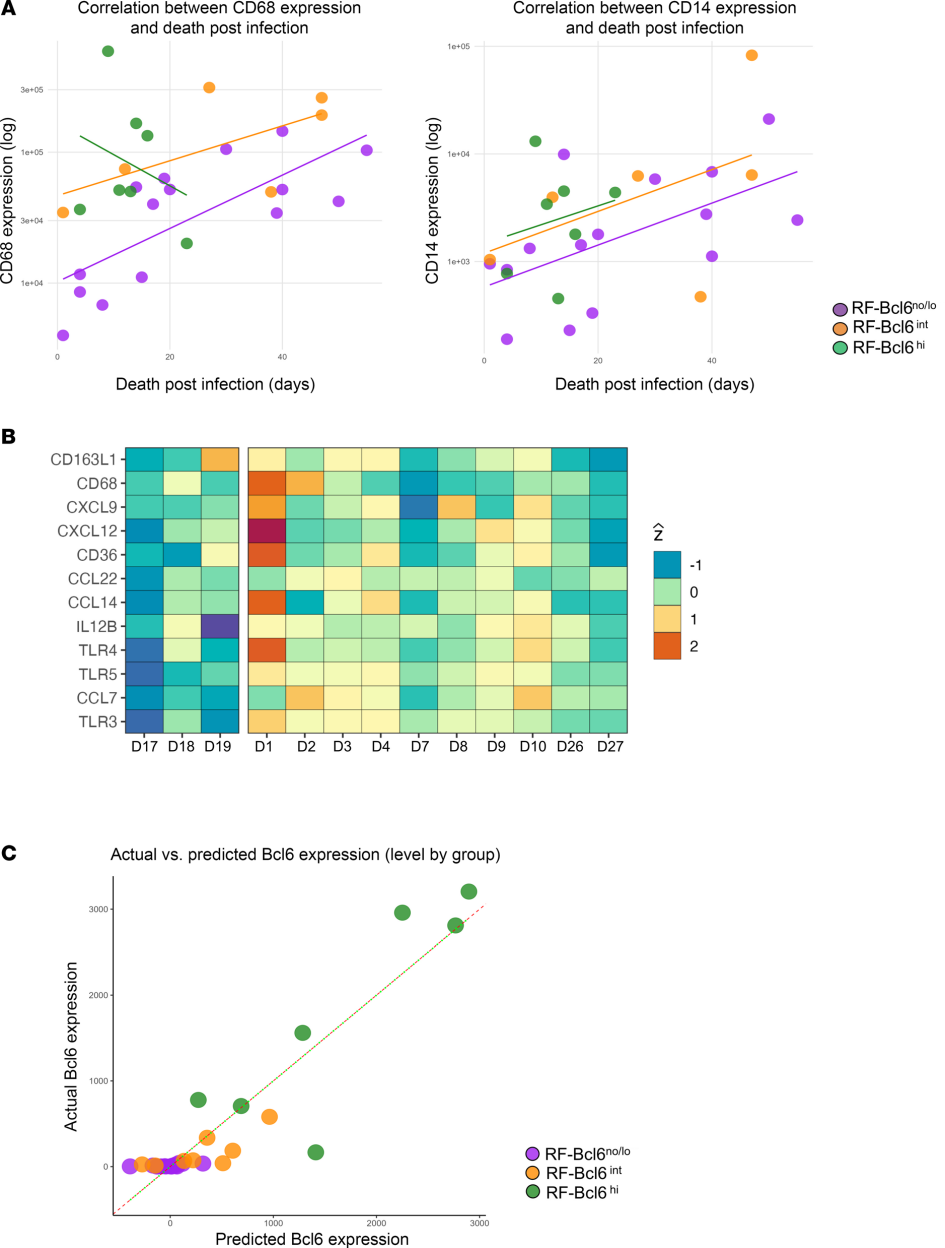
RF-Bcl6^no/lo^ follicles exhibit a distinct in situ macrophage profile in COVID-19 LD-LNs. (**A**) Left panel: Linear regression analysis of log-transformed CD68 expression versus days post-infection (DPI) in LD-LNs from COVID-19–infected individuals. Right panel: Linear regression analysis of log-transformed CD14 expression versus DPI. Log expression levels of CD68 and CD14 are plotted on the *y* axis, and DPI on the *x* axis. Data points represent individual donors: RF-Bcl6^no/lo^ (purple circles), RF-Bcl6^int^ (orange circles), and RF-Bcl6^hi^ (green circles). Regression lines illustrate group-specific trends. Statistical results for CD68 and CD14 are summarized in Supplemental Table 9. (**B**) Heatmap showing macrophage-specific differentially expressed genes (DEGs) in RF-Bcl6^no/lo^ versus RF-Bcl6^hi^ individuals. The *y* axis lists macrophage-related genes, and the *x* axis displays individual donors, grouped by RF-Bcl6^hi^ (left) and RF-Bcl6^no/lo^ (right). Colors represent Z-scored expression values. (**C**) Multiple linear regression analysis comparing predicted versus observed Bcl6 expression levels. Data points are color-coded by group: RF-Bcl6^no/lo^ (purple), RF-Bcl6^int^ (orange), and RF-Bcl6^hi^ (green). The dashed line represents a perfect correlation between predicted and actual values. Supplemental Table 10 summarizes the regression model, including marker predictors, estimated coefficients, standard errors, t-values, and *P* values. Significant associations were found for CD20 Ki67 (positive predictor) and CD68 (negative predictor).

Next, we investigated the prevalence of innate immunity subsets (CD68, CD14 for monocytes/macrophages, and MPO, a marker of granulocytes) for all available tissues (Supplemental Figure 9A). Since the innate antibody panel (Supplemental Table 3) did not include follicular markers, our histocytometry analysis was applied to both F and EF areas (Supplemental Figure 9B). We observed a significant association between CD68 (*P* = 0.002) (Figure 7A and Supplemental Table 9) or CD14 (*P* = 0.024) (Figure 7A and Supplemental Table 9). Histocytometry-calculated counts and death postinfection (DPI) in RF-Bcl6^no/lo^ donors (CHUV site), suggesting a link between follicular monocytes/macrophages and disease severity and clinical outcome.

To gain further insight into the potential role of monocyte/macrophages, we further investigated macrophage-associated gene expression across tissue groups. Using our GeoMx dataset, we selected genes related to RF macrophage activity (e.g., *IL12B*, *CXCL9*, *CCL7*, *CD163L1*, and *CD68*) and compared their expression levels between groups. An overall upregulation of these genes was found in RF-Bcl6^no/lo^ compared with RF-Bcl6^hi^ (Figure 7B), suggesting a more pronounced macrophage-driven inflammatory response in the absence of robust GC formation. To further investigate the link between macrophages and Bcl6 expression, we built a multiple-regression model using total cell counts from our histocytometry data, integrating both adaptive and innate panels. Major immune populations were included (CD20^hi/dim^, CD20^hi/dim^Ki67^hi^, PD1^hi^, PD1^hi^Ki67^hi^, CD8^hi^, CD8^hi^GrzB^hi^, CD14^hi^, CD68^hi^, and MPO^hi^), excluding those directly involving Bcl6 staining. The model showed high predictive accuracy, with predicted Bcl6 expression closely matching observed levels across RF-Bcl6 groups (Figure 7C). Regression analysis revealed a strong positive association between Bcl6 and CD20^hi/dim^Ki67^hi^ levels (β = 0.124, *P* = 2.3 × 10^–6^) and a significant negative association with CD68^hi^ expression (β = –0.002, *P* = 0.012) (Supplemental Table 10), suggesting that macrophage infiltration may suppress Bcl6 expression. These findings support a model in which elevated macrophage levels in RF-Bcl6^no/lo^ tissues could limit GC responses.

## Discussion

Given the limited accessibility to relevant human LNs, the cellular and molecular mechanisms regulating follicular/GC immune dynamics in draining LNs during viral infections, particularly in the acute phase, are poorly understood. Here, we leveraged LD-LNs and matched distal LNs from COVID-19 autopsies to investigate the immune landscape of RFs and the molecular pathways active in situ. Additionally, we included 4 HLN-Control tissues from non-COVID-19 donors as a healthy reference baseline.

We stratified COVID-19 LD-LNs into 3 distinct groups based on Bcl6 expression profile to assess follicular immune reactivity. By analyzing CD20^hi/dim^ expression, we confirmed that all RFs displayed Ki67 expression, indicating active proliferation and confirming these structures as secondary follicles rather than primary or resting (nonproliferative) follicles. Based on the visual inspection of individual tissues and the profile of the measured Bcl6^hi^CD20^hi^ B cell counts per follicular area and donor, 3 LD-LNs groups (RF-Bcl6^no/lo^, RF-Bcl6^int^, and RF-Bcl6^hi^) were defined. We found a substantial variability of Bcl6 prevalence among individual RFs from the same donor, both in the RF-Bcl6^int^ and RF-Bcl6^hi^ groups.

In the RF-Bcl6^no/lo^ group, we observed smaller RF areas, reduced levels of proliferating B cells (CD20^hi/dim^Ki67^hi^) compared with the other 2 groups, and a distinct FDC staining pattern as previously reported (12), collectively suggesting that these structures represent less mature rather than resting RFs. Additionally, we observed inconsistent coexpression of CD4 and CD57 in RFs, likely due to CD4 downregulation in PD1^hi^CD57^hi^ Tfh cells. Thus, we analyzed PD1^hi^CD57^hi^ and PD1^hi^CD57^lo^ cells as surrogate markers for Tfh cells. The higher PD1 expression in RFs compared with EF areas, combined with the significantly lower PD1 expression in CD8^hi^ T cells, supports the accuracy of our Tfh cells quantification (13).

By incorporating B cell and CD4 T cell subset cell densities, we observed a distinct clustering between HLN-Controls of non-COVID-19–infected and COVID-19 LD-LNs. This was the case even between HLN-Controls and RF-Bcl6^no/lo^ LNs, 2 groups with comparable Bcl6 expression. Therefore, COVID-19 induces a unique LD-LN immune reactivity, regardless of the Bcl6 expression. Among the COVID-19 donors, RF-Bcl6^hi^ tissues were separately clustered, while RF-Bcl6^no/lo^ and RF-Bcl6^int^ tissues showed overlapping clustering patterns. This suggests that RF-Bcl6^int^ may represent a transitional stage between RF-Bcl6^no/lo^ and RF-Bcl6^hi^ phenotypes.

Despite their similar cell densities of CD20^hi/dim^Ki67^hi^ and PD1^hi^ cells, RF-Bcl6^int^ and RF-Bcl6^hi^ LD-LNs showed different spatial distribution of B cell and Tfh cell subsets. The PD1^hi^CD57^hi^ phenotype marks a functionally distinct subset of Tfh cells (positioned closer to the dark zone) (9) with a unique molecular profile (9, 14, 15). We observed a clear trend for closer proximity between PD1^hi^CD57^hi^ Tfh cells and CD20^hi/dim^Ki67^hi^ B cells in RF-Bcl6^hi^ tissues compared with HLN-Controls and RF-Bcl6^int^, suggesting increased B/T cell interactions in this group. The spatial positioning of these subsets further supports the hypothesis that RF-Bcl6^int^ represents an intermediate or less mature stage of RF development, while RF-Bcl6^hi^ reflects a more established GC phenotype.

Direct comparison between COVID-19 LD-LNs, matched SD-LNs, and distal LNs revealed that COVID-19 may not induce a generalized RF Bcl6 immune reactivity in LNs from different anatomical sites. Despite widespread inflammation and potential systemic immune activation (16), mature RFs were selectively induced in lung-draining lymphoid organs. This suggests that Bcl6-dependent GC formation in COVID-19 could be influenced by the local availability of antigens or local inflammatory signals. The formation of tertiary lymphoid structures (TLS) in long COVID-19 has been described (17), but whether RF-Bcl6^hi^ reactivity in LD-LNs is linked to lung-associated TLS in our cohort remains to be determined.

Among serum markers, CRP levels were higher in RF-Bcl6^no/lo^ compared with RF-Bcl6^hi^ tissues, but this profile was not associated with lymphopenia or elevated neutrophil counts, which are hallmarks of severe hyperinflammation in COVID-19 (18). However, our modeling analysis found that Bcl6 expression in LD-LNs was not associated with time after infection (DPI), suggesting that the distinct RF patterns observed were driven by individual immune responses rather than simply the duration of infection (6).

Our transcriptomic analysis identified distinct molecular signatures associated with RF-Bcl6 expression levels. In line with previous studies, the absence of Bcl6 expression in COVID-19 hilar LNs has been linked to TNFA overexpression, which impairs Tfh differentiation. Our data extend previous findings by demonstrating that compromised Bcl6 expression in LD-LNs is associated with an upregulation of TH1-favoring and TNF-related genes within follicular/GC areas. Specifically, genes known to inhibit Tfh development, such as *KLF2* (19), *STAT4* (20), *STAT5A/B* (21), *IFNAR1*, and *TGFB3* (22), were enriched in RF-Bcl6^no/lo^ tissues. Additionally, genes characteristic of a TH1-response, including *CXCR3*, and TNF-related genes such as TNF-receptors (*TNFRSF1A*, *TNFRSF25*, *TNFRSF10B*) and TNF-signaling mediators (e.g., *TRAF2*), were also elevated. Other key regulators of TNF signaling, including *TNFRSF1B* and *TNFRSF10C*, which are involved in immune modulation and apoptosis pathways, were significantly overexpressed in RF-Bcl6^no/lo^ tissues compared with RF-Bcl6^hi^ tissues.

Conversely, RF-Bcl6^hi^ tissues showed significant enrichment in genes favoring B/Tfh development/trafficking, GC maintenance, and T cell development (e.g., *BCL6*, *AICDA*, *CD74*, *STAT3*, *CXCL13*, *S1PR*, and *TCF3*). RF-Bcl6^int^ tissues displayed an intermediate transcriptional profile, reinforcing the idea that these follicles represent a transitional state.

Our cell deconvolution approach does not assign the expression of a given gene to a specific cell type per se; rather, it indicates the relative association between gene expression and the abundance or not of the cell type. We found either none or a negative association between TNF-family genes and B cells or Tfh cells, indicating that possibly other cell types are the main contributors of these genes in the RF-Bcl6^no/lo^ group. Our analysis cannot assess the contribution of stromal cells like FDC or macrophages that presumably could play a major role in TNF signaling (23). Interestingly, a different profile was found for the RF-Bcl6^hi^ group, where the *TNFR2/TRAF2* axis and decoy receptors (*DR1*, *DR3*) positively associated with B cells. The data indicate that the TNF-related signaling network could have a differential role in follicular reactivity, mediated by the different cell types responding to TNF signals, with respect to Bcl6 expression.

Total CD68 and CD14 prevalence increased over time after infection in RF-Bcl6^no/lo^ tissues only, suggesting that macrophage accumulation and prolonged inflammation could contribute to sustained TNF-driven immune regulation. This aligns with previous findings showing that TNFA from macrophages is a key factor in the loss of GC Bcl6 expression in COVID-19 LNs (6). In line with this, our spatial transcriptomic analysis revealed a higher in situ expression of follicular macrophage function–related genes in RF-Bcl6^no/lo^ tissues. We should emphasize, though, that our approach cannot assign this gene signature to a specific monocyte/macrophage or stromal cell type. The cellular source of such gene expression needs further investigation. Additionally, our predictive model for Bcl6 expression identified CD68 as a significant negative predictor. This suggests that an inflammatory macrophage-rich environment may counteract the establishment of Bcl6-dependent GC structures, potentially through excessive TNF signaling and dysregulated immune interactions (24).

Our study provides a comprehensive cellular and molecular analysis of RF dynamics in COVID-19 LD-LNs, identifying distinct immune microenvironments in RF-Bcl6^hi^ and RF-Bcl6^no/lo^ tissues. Individuals exhibited different RF patterns, reflecting variations in GC activity, immune composition, and in situ operating inflammatory molecular signaling. While RF-Bcl6^hi^ tissues sustained active GC formation, RF-Bcl6^no/lo^ tissues exhibited TNF-driven immune dysregulation, impairing Tfh cell differentiation and B cell maturation.

While our study is limited by the absence of antibody data, it nonetheless yields meaningful insights. The inclusion of non–COVID–19 controls revealed a distinct follicular immune cellular composition between RFs in COVID-19 and control tissues. Whether this is a profile that applies to other acute viral infections needs to be addressed. Overexpression of the identified TH-1–related pathways, as well as their interplay with other inflammatory pathways (e.g., type I IFNs), as a potential negative regulator of follicular/GC immune reactivity in chronic infections (e.g., HIV) or lymphomas, should be further investigated, at least in situ, using relevant tissues. Therefore, our findings offer a valuable foundation for future research on follicular immunity, especially given the difficulty of accessing human LNs during acute infection. To this end, the application of complementary methodologies like multiplex imaging and spatial biology, and the analysis of generated data with advanced computational tools, is of great importance. Such analysis can provide a high-dimensional, comprehensive profiling of relevant tissues and result in the identification of tissue signatures that could benefit our knowledge regarding the immunobiology of follicular cells in a given disease.

## Methods

### Sex as a biological variable.

This study includes both male and female participants. Due to the limited sample size, sex was not treated as a covariate in the analysis. The cohort consisted of 22 male donors and 14 female donors.

### Study cohort and tissue samples.

LN tissues were obtained from the Institute of Pathology at Lausanne University Hospital and the Feinberg School of Medicine at Northwestern University (see Table 1 and Supplemental Table 1). Postmortem samples from patients who died of COVID-19 were collected at: (a) the Institute of Pathology, CHUV, between March 2020 and March 2021, and (b) the Department of Pathology, Northwestern University, between March 2020 and July 2021. HLN-Control tissues were sourced from Northwestern University. C-reactive protein (CRP) levels were measured using an immunoturbidimetric assay. Absolute counts of leukocytes, lymphocytes, neutrophils, monocytes, eosinophils, and basophils (expressed as G/L) were obtained using an automated hematology analyzer.

### LN sampling and anatomical localization.

LNs from USA cohort (*n* = 10) were collected from thoracic (paratracheal, mediastinal, paraesophageal, paraaortic, and hilar) and axillary sites. This cohort was subdivided into: RF-Bcl6^no/lo^ (*n* = 7; paratracheal, mediastinal, axillary, paraesophageal, and paraaortic) and RF-Bcl6^int^ (*n* = 3; mediastinal and paraaortic). LNs from Switzerland cohort (*n* = 22) included LNs from the hilar and subdiaphragmatic regions. These were further classified as: RF-Bcl6^no/lo^ (*n* = 11; primarily hilar, with 3 donors contributing both hilar and subdiaphragmatic nodes), RF-Bcl6^int^ (*n* = 5; hilar and subdiaphragmatic/hilar), and RF-Bcl6^hi^ (*n* = 7; hilar and subdiaphragmatic/hilar). Previous reports have shown functional similarities between hilar and mediastinal LNs, justifying their joint analysis in thoracic immune studies (25, 26). Control hilar LNs (HLN-Controls, *n* = 4) from non–COVID-19 individuals were also analyzed, comprising 3 paratracheal and one lung hilum sample.

### mIF.

mIF staining was performed on 4 μm–thick formalin-fixed, paraffin-embedded (FFPE) tissue sections mounted on Superfrost glass slides (Thermo Scientific). To facilitate deparaffinization, slides were first placed on a hot plate at 69°C for 30 minutes. The staining protocol was fully automated using the Ventana DISCOVERY ULTRA system (Roche Diagnostics), beginning with 3 deparaffinization cycles at 69°C, each lasting 8 minutes. Antigen retrieval was performed using DISCOVERY Cell Conditioning Solution 1 (CC1; Tris-EDTA buffer, pH 8.0; Roche Diagnostics, catalog 06414575001) at 95°C for 48 minutes to unmask epitopes. This was followed by incubation with DISCOVERY Inhibitor (Roche Diagnostics, catalog 05279798001) for 8 minutes to block endogenous peroxidase activity and prevent nonspecific staining. For each target, the protocol involved the sequential application of titrated primary antibodies, followed by HRP-conjugated secondary antibodies and Opal tyramide signal amplification (TSA) dyes (Akoya Biosciences). After each Opal application, antibody stripping was performed using DISCOVERY Cell Conditioning Solution 2 (CC2; citrate buffer, pH 6.0; Roche Diagnostics, catalog 05279798001) at 95°C for 8 minutes to ensure complete removal of previous antibodies and prevent cross-reactivity. Following the final staining step, sections were counterstained with Spectral DAPI (Akoya Biosciences, catalog FP1490) to visualize nuclei. To ensure thorough reagent removal, slides were washed using a mild detergent solution (Roche Diagnostics) followed by several rinses in distilled water. Slides were then mounted using Dako Fluorescence Mounting Medium (Agilent Technologies, catalog S3023), optimized to preserve fluorescence signals and minimize photobleaching during imaging. Stripping efficiency was validated through spectral unmixing in the imaging software Phenochart 1.0.12 (Akoya), ensuring that detected fluorescence originated from specific staining rather than autofluorescence or antibody carryover in the single-cycle staining process.

### GeoMx digital spatial profiling (DSP).

Spatial transcriptomic profiling was performed using the GeoMx DSP platform (NanoString) with the Human Whole Transcriptome Atlas, targeting approximately 18,000 genes. FFPE tissue sections (4 μm) were prepared from *n* = 15 RF-Bcl6^no/lo^, *n* = 6 RF-Bcl6^int^, and *n* = 3 RF-Bcl6^hi^ LNs. Regions of interest (ROIs) were manually selected from B cell follicles based on corresponding multiplex immunofluorescence images using the following fluorescent markers: FITC/525 nm (CD3); Cy3/568 nm (Syto83); Texas Red/615 nm (CD45) and Cy5/666 nm (CD68). Between 9 and 12 ROIs per tissue were selected to maintain consistent area across samples (average area: 245,745.52 μm²). Data acquisition was performed in 2 independent experiments (A and B). Raw sequencing counts from Digital Count Conversion (DCC) files were extracted, and barcode mappings from Probe Key Code (PKC) files were matched to gene targets. Donor metadata were integrated to assign each ROI to its original group.

Although both Swiss and US samples were used for normalization and batch correction, only Swiss tissues were retained for downstream analyses and visualizations such as PCA. This approach preserved data integrity and ensured consistent batch correction across the full dataset, while restricting biological comparisons to the target population.

### Statistics.

An unpaired nonparametric Mann-Whitney *U* test was used for mIF data analysis, including comparisons of cell markers and quantification of follicles analyzed per donor across RF-Bcl6 reactivity groups. A Mann-Whitney *U* test was also applied to evaluate the time interval between death and autopsy across groups. Wilcoxon signed-rank test was used for paired comparisons (LD-LNs versus SD-LNs), and a linear mixed-effects model was used for serological data with fixed effects for group comparisons and random effects for individual donors. All statistical tests were corrected for multiple comparisons using the Benjamini-Hochberg FDR procedure to control the expected proportion of false positives among significant results. This adjustment was particularly important for our datasets, where multiple hypotheses were tested simultaneously. Only raw *P* values are displayed in the figures, with *P* ≤ 0.05 considered significant. The complete summary of statistical analysis for cell comparisons, including both raw and FDR-adjusted *P* values with significance indicators for main and supplemental figures, is provided in Supplemental Table 11. Graphs were produced using GraphPad Prism (v8.3.0) and RStudio (v4.4.2).

### Study approval.

The studies were approved by the Ethical Committee of (a) the Canton de Vaud, Switzerland (protocol no. 2020-01257) and (b) Northwestern University (STU 00202918). Written consent was obtained from all living participants, and for samples previously donated to tissue repositories from deceased patients, an IRB-approved waiver of consent was applied (Northwestern University, CHUV). The use of HLN-Controls was approved by Northwestern University (STU 00202918). This research project was conducted according to the principles of the Declaration of Helsinki.

### Data availability.

The authors agree to share all publication-related data. FASTQ files generated from the GeoMx experiments are publicly available on the European Nucleotide Archive (ENA) under the study accession no. PRJEB89778. The presented data are accessible through https://doi.org/10.5281/zenodo.15536459 Values for all data points in graphs are reported in the Supporting Data Values file.

## Author contributions

CB and KI performed experiments and image analysis. SG and MO contributed to image analysis. CB drafted the manuscript. JB supervised the SQL clustering analysis. BBM and RG performed and supervised the spatial transcriptomic analysis. CB participated in the transcriptomic analysis. S Burgermeister and CB performed neighboring analysis. OYC supervised the statistical analysis. MLS and S Berezowska assisted with the clinical data, serological measurements, and pathological evaluation/information. NS provided serological measurements. MJF, JWL, and LDL provided tissue material and pathological information. GP assisted with the interpretation of the immunological data and the manuscript preparation. CP conceived, designed, and supervised the study, interpreted data, and edited the manuscript. YDM provided clinical information and assisted with the clinical data interpretation. All authors have read, edited, and approved the final version for submission.

## Funding support

- Swiss National Science Foundation (SNF, 310030_204226 to CP and 196852 to GP)
- Institute of 574 Pathology, Department of Laboratory Medicine and Pathology, Lausanne University
- 575 Hospital and Lausanne University, Lausanne, Switzerland

## Supplementary Material

Supplemental data

Supporting data values

## Figures and Tables

**Table 2:**
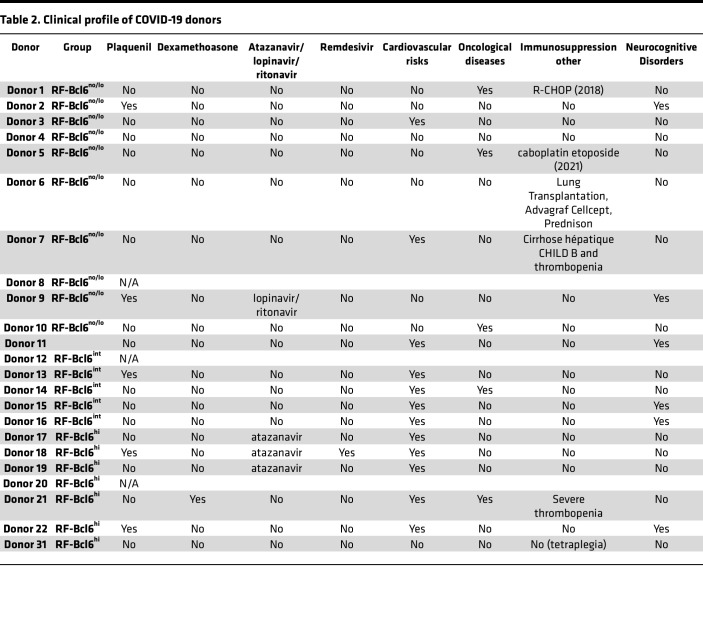
Clinical profile of COVID-19 donors

**Table 1:**
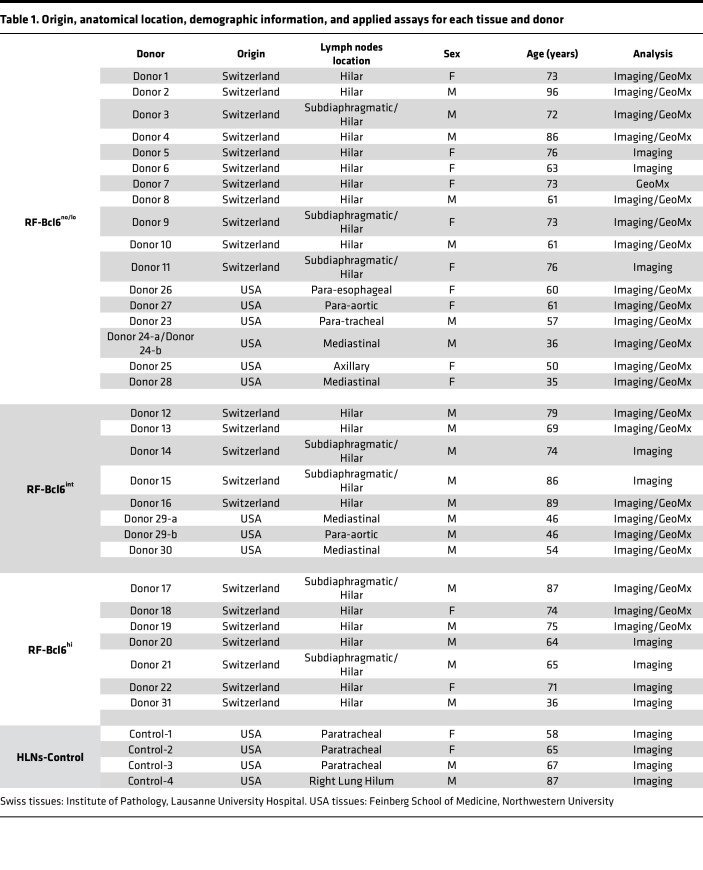
Origin, anatomical location, demographic information, and applied assays for each tissue and donor

## Version 1 09/09/2025

In-Press Preview

## Version 2 10/22/2025

Electronic publication
