# Supplementary material for: Bcl6 expression is associated with a distinct immune landscape and spatial transcriptome in COVID-19
Source: JCI Insight. 2025 Sep 9;10(20):e189134. doi: 10.1172/jci.insight.189134 (PMC12581676; doi:10.1172/jci.insight.189134)
Supplement: Supplemental data [file jciinsight-10-189134-s165.pdf]

Antibodies panel and dilution. Two multiplex panels were designed to assess adaptive and innate immunity, along with a third panel for follicular dendritic cells (FDCs). Antibodies were carefully titrated on control tissue sections to optimize specificity and signal intensity. Fluorophores were assigned based on spectral compatibility to minimize crosstalk between channels. Antibody dilutions were adjusted according to antigen expression levels, with lower dilutions for low-abundance markers and higher dilutions for more abundant targets to maintain an optimal signal-to-noise ratio. The antibodies were applied in the following order for the experiment: in the adaptive immunity panel, PD-1 (1:100, Opal 620), CD57 (1:300, Opal 570), Bcl-6 (1:150, Opal 480), CD20 (1:400, Opal 520), CD4 (1:200, Opal 690), Ki67 (1:300, TSA-DIG and Opal 780), and DAPI. The innate immunity panel included GrzB (1:40, Opal 570), CD8 (1:100, Opal 690), CD14 (1:50, Opal 620), MPO (1:150, Opal 520), and CD68 (1:200, Opal 780). The FDC panel consisted of FDC (1:200, Alexa 488) and CD20 (1:45, conjugated-eFluor 650). For more details on antibody references, catalog numbers, concentrations, and company information, refer to **Supplemental Table 2 and Supplemental Table 3**.

Imaging data acquisition. Multispectral images (MSI) were acquired using i) the Vectra Polaris 1.0 imaging system (Akoya) at a resolution of 5 μm/pixel (20×) and ii) the Leica Stellaris 8 SP8 confocal system, equipped with LAS-X software at 512 × 512-pixel density (20× objective). A compensation matrix was generated using the Leica LAS-AF Channel Dye Separation module (Leica Microsystems) and single-stained tissue sections to correct for fluorophore spillover.

Morphological characteristics of follicular areas. Morphological analysis of individual follicular ROIs, identified based on the density of CD20$^{high/dim}$ B-cells, was carried out using FIJI software, where various features such as the area, circularity, and solidity were extracted. To ensure robust analysis, approximately 20 ROIs per tissue were meticulously chosen, each

representing distinct and well-defined follicular structures within the tissues. Subsequently, from each ROI, follicle areas were extracted and converted into μm^2 using the tissue scale bar. This scaling process ensured accurate representation of follicle sizes in absolute units, enabling meaningful comparisons across samples.

Classification of RF-Bcl6 expression groups. Donors were classified into three RF-Bcl6 expression groups (RF-Bcl6$^{no/low}$, RF-Bcl6^{int}, and RF-Bcl6^{high}), based on quantitative Bcl6 expression within the follicular areas of LD-LNs. The classification was determined by analyzing the distribution of Bcl6^{high} B-cell counts per follicular area and per total follicular area within each sample. Thresholds were manually defined based on the observed distribution patterns, supported by Histocytometry-based quantification and visual inspection of mIF images. This combined approach ensured that the assigned groups reflected biologically meaningful and histologically distinct patterns of Bcl6 expression.

Distance analysis and spatial distribution. To investigate the spatial relationship between GC B-cells and T_{FH}-cells, the minimum Euclidean distance between individual B-cells and their nearest T_{FH} neighbor was computed within each follicle containing at least 20 positive cells per population. Analyses were performed in R version 4.4.2, using the "spatstat", "ggplot2", and "readxl" packages. The interaction matrices were generated from X and Y coordinates of each cell type, and mean distances were extracted for each follicular area (ROI). Spatial organization patterns were further assessed using Ripley's G-function, comparing the observed distribution to a theoretical Poisson distribution (CSR: complete spatial randomness). The area under the observed and theoretical curves was calculated to quantify clustering (AUC_{above}) or dispersion (AUC_{below}). Net deviation ($AUC_{above} – AUC_{below}$) was used to summarize the spatial proximity. Results were visualized as dot plots (mean minimum distance per follicle) or as bar graphs showing distance distribution histograms with overlaid density curves. Connectivity plots were generated to map spatial links between the most central GC B-cell and surrounding T_{FH}-cells.

All spatial analyses and visualizations were carried out using custom scripts in R [https://github.com/Clob9100/DistanceAnalysisSpatial].

GeoMx data processing and quality control. We analyzed NanoString DSP data using standard GeoMx processing workflows (1) and R version 4.3.2. Specifically, we processed the DCC files and conducted quality control at the segment, probe, and gene levels using the R packages "GeoMxWorkflows" (1). "GeomxTools" (2), and "NanoStringNCTools" (3). First, we adjusted all zero expression counts to one to enable subsequent data transformations. We then implemented several quality control metrics recommended by NanoString for our segments, including a minimum of 1000 reads, 80% trimming, stitching, and alignment, 50% sequencing saturation, a minimum negative control count of 1, a maximum of 1000 reads observed in NTC wells, and a minimum area of 1000. Next, we removed probes for which the average count across segments was less than 10% of the average count for all probes targeting the same gene across segments, as well as probes that were outliers in at least 20% of the segments. Finally, we filtered out segments and genes with low signal, specifically removing segments where less than 5% of panel genes were detected above the level of quantification (LOQ, defined as two standard deviations above the mean) and genes detected below the LOQ in at least 10% of segments.

GeoMx batch correction. To perform batch correction, we first normalized the raw data using the Trimmed Mean of M-values (TMM) method with the R package "standR" (4), which adjusts for differences in library sizes and composition between RNA-seq samples. We then applied the RUV-4 correction from the same package (5), which removes unwanted variations by identifying negative control genes and calculating scaling factors for batch correction. In our analysis, we identified 300 negative control genes and set the number of scaling factors to 2.

GeoMx differential gene expression and enrichment analysis. To perform differential gene expression, we followed the limma-voom pipeline (6). We defined a linear model with a design matrix containing the treatment variable and weight matrix from the RUV-4 correction method as covariates. Using this model, we then compared every pair of treatments, identifying differentially expressed genes as those presenting an adjusted p-value smaller than 0.05. Alternatively, we also studied the differences across the different treatments using the R package DESeq2 (7), comparing the intercepts for every treatment in a negative binomial regression, also accounting for the scaling factors from the RUV-4 model. Using the differentially expressed genes in each comparison, we then performed enrichment analysis (GSEA) to identify relevant biological pathways (8). To do so, we employed the "fry" method from the R package limma (6), using the canonical pathways gene sets from the Reactome pathway database (9). The results were then analyzed and visualized using the R package vissE (10).

Cell deconvolution. We used the "SpatialDecon" R package (11) to conduct cell deconvolution on the GeoMx dataset. To do so, we took single-cell RNA-seq data from HIV patients as our reference dataset deposited in the GEO database under the accession ID GSE288212. Our analyses used a broad classification of cells, which included B-cells and T_{FH}-cells. We restricted our gene selection to those expressed in immune and stromal cells, using the "safeTME" cell profile matrix from "SpatialDecon" along with a set of genes relevant to T_{FH} differentiation, B-cell help, and associated immune regulation(e.g., *IL-17A, BCL6, CXCR5, IL-4, IL-21, IL-6, IL-1B, PDCD1, ICOS,* and *IL-10*). To minimize potential effects from cell population size differences and genetic heterogeneity within cell types, we first applied a k-means clustering approach with 15 centers to classify each cell type into more refined subpopulations based on their gene signatures (12). These newly defined subpopulations were then used to construct a lymph node-specific cell profile matrix, which served as input for "SpatialDecon" to perform

the deconvolution (11). This provided us with the cell proportion of every cell subpopulation for each segment. To obtain the cell proportions of the broad cell classification, we summed across the different cell subpopulations. For downstream analysis of the deconvolution results, we used the “DESeq2” R package (7) to model gene expression counts using a negative binomial regression. As explanatory variables, the model included the RUV-4 scaling factors, segment area, treatment, and the results of the deconvolution for a given cell population. Specifically, we used a model with the treatment effect and treatment-specific slopes for each cell percentage. Note that each cell population was modelled independently, and that the percentage values were scaled before including them in the model. This model helped us assess the relationship between the proportion of a particular cell type in a given segment and the expression levels of specific genes. We analyzed the proportion of T_{FH}-cells relative to the proportion of B-cells.

SQL and PCA projection for data visualization. We used the Statistical Quantile Learning (SQL) method (13), a tool for nonlinear dimensionality reduction, to summarize, analyze, and visualize adaptive and innate immunity data separately [https://github.com/jbodelet/SQL]. We chose SQL due to its capability to handle high-dimensional datasets and accurately capture complex, nonlinear relationships often found in biological data. The adaptive immune panel featured cell subsets such as $CD20^{high/dim}$, $CD20^{high/dim}Ki67^{high}$, $CD20^{high/dim}Ki67^{high}Bcl6^{high}$, $CD4^{high}$, $PD1^{high}$, $PD1^{high}Ki67^{high}$, $PD1^{high}CD57^{high}$ and $PD1^{high}Ki67^{high}Bcl6^{high}$. These markers were specifically chosen to aid in clustering and classifying our immune data and were extracted from our Histocytometry analysis.

The SQL method provides a straightforward approach to estimating nonlinear latent variable models, or generative models. Unlike local methods such as UMAP, SQL assumes a probabilistic model and learns a generator, which is a smooth function that connects the latent space to the data space. This approach enables SQL to learn a global latent space that captures

the overall structure of the data, whereas UMAP emphasizes preserving local neighborhood relationships. The generator allows for easy reconstruction of the data from the latent space, which permits interpretability of the latent space. Compared to other generative methods (such as Variational Autoencoders), SQL is not only easy to fit but also performs better for small samples and large-dimensional data. SQL provides statistical guarantees and interpretable latent variables, making it a reliable tool for uncovering insights that other methods might miss.

We applied this approach to cluster the four groups—RF-Bcl6$^{no/low}$, RF-Bcl6^{int}, RF-Bcl6^{high}, and HLN-Controls. This method enhanced group separation and provided deeper insights into adaptive and innate immunity across age groups, improving the understanding of immune response variations.

For the spatial transcriptomic data, Principal Component Analysis (PCA) was employed to analyze specific gene sets across the section ROIs. This technique reduces the high-dimensional gene expression data into principal components that capture the most significant variance, facilitating a clearer visualization of patterns across the different ROI groups.

Imaging data analysis. For the Vectra Polaris-generated images, the Phenochart 1.0.12 software (Akoya), a whole-slide viewer for high-resolution multispectral acquisition and annotation capabilities, was used to navigate among slides and identify specific Regions of Interest (ROIs). The subsequent imaging and analysis were performed on the whole tissue. The acquired MSI were analyzed using the InForm analysis software, version 2.4.8 (Akoya). Initially, the images were unmixed, and specific ROIs were used for the algorithm training and cell segmentation across the imaged tissue. The segmentation process involved utilizing the appropriate training components, such as CD20, BCL6, and PD1, to define the GC, Extrafollicular area (EF), and region without tissue. Tissue segmentation was based on selected training markers and DAPI expression, extracting the autofluorescence signal of the tissue. This

was achieved by manually drawing training regions for each analyzed image. The segmentation algorithm was trained individually on each image, ensuring a training accuracy of over 90% for the ROIs segmentation. Next, individual cells were segmented using an adaptive cell segmentation algorithm based on the counterstained sections. Cytoplasm and membrane markers were utilized to help in this cell segmentation process. A file report was generated, containing the spatial coordinates (X, Y) of each segmented cell, along with their mean intensity for each Opal fluorophore used. This file report was then extracted and converted into a FACS file (FSC) format before being uploaded to the FlowJo 10 software and the generated data were further analyzed with HistoFlowCytometry (14). Data are reported as normalized numbers per μm^2 or frequency of total imaged cells.

References

1. Reeves J, DP ON GM, Yang Z, Zimmerman S, and Vitancol R. GeoMxWorkflows: GeoMx digital spatial profiler (DSP) data analysis workflows. *R package version.* 2023;1(0).
2. Ortogero N, Yang Z, Vitancol R, Griswold M, and Henderson D. GeomxTools: NanoString GeoMx Tools. *R package version.* 2022;1(0).
3. NanoStringNCTools AP. NanoString nCounter Tools. *R package version.* 2023;1(1).
4. Liu N, Bhuva DD, Mohamed A, Bokelund M, Kulasinghe A, Tan CW, et al. standR: spatial transcriptomic analysis for GeoMx DSP data. *Nucleic Acids Research.* 2024;52(1):e2-e.
5. Gagnon-Bartsch JA, Jacob L, and Speed TP. Removing unwanted variation from high dimensional data with negative controls. *Berkeley: Tech Reports from Dep Stat Univ California.* 2013:1-112.
6. Ritchie ME, Phipson B, Wu D, Hu Y, Law CW, Shi W, et al. limma powers differential expression analyses for RNA-sequencing and microarray studies. *Nucleic acids research.* 2015;43(7):e47-e.
7. Love MI, Huber W, and Anders S. Moderated estimation of fold change and dispersion for RNA-seq data with DESeq2. *Genome biology.* 2014;15:1-21.
8. Subramanian A, Tamayo P, Mootha VK, Mukherjee S, Ebert BL, Gillette MA, et al. Gene set enrichment analysis: a knowledge-based approach for interpreting genome-wide expression profiles. *Proceedings of the National Academy of Sciences.* 2005;102(43):15545-50.
9. Bhuva D, Smyth G, and Garnham A. Msigdb: an ExperimentHub package for the molecular signatures database (MSigDB). *R package version.* 2023;1(0).
10. Bhuva D. vissE: visualising set enrichment analysis results. *R package version.* 2021;122.
11. Danaher P, Kim Y, Nelson B, Griswold M, Yang Z, Piazza E, et al. Advances in mixed cell deconvolution enable quantification of cell types in spatial transcriptomic data. *Nat Commun.* 2022;13(1):385.
12. Lun AT, McCarthy DJ, and Marioni JC. A step-by-step workflow for low-level analysis of single-cell RNA-seq data with Bioconductor. *F1000Res.* 2016;5:2122.
13. Bodelet, J., Blanc, G., Shan, J., Muniz Terrera, G., & Chén, O. Y. (2025). Statistical Quantile Learning for Large Additive Latent Variable Models. *Journal of the American Statistical Association*, 1–22. https://doi.org/10.1080/01621459.2025.2526697.
14. Gerner MY, Kastenmuller W, Ifrim I, Kabat J, and Germain RN. Histo-cytometry: a method for highly multiplex quantitative tissue imaging analysis applied to dendritic cell subset microanatomy in lymph nodes. *Immunity.* 2012;37(2):364-76.
